# A Rare Case of Invasive Central Nervous System Aspergillus niger in a Previously Immunocompetent Patient After Corticosteroid Treatment for COVID-19

**DOI:** 10.7759/cureus.57923

**Published:** 2024-04-09

**Authors:** Hussein Saleh, Peter Abraham, Hassan Chahine, Shanmuga Subbiah, Nadine Grace-Abraham

**Affiliations:** 1 College of Osteopathic Medicine, Michigan State University, East Lansing, USA; 2 Radiology, University of California San Diego, San Diego, USA; 3 Internal Medicine, Emanate Health, West Covina, USA; 4 Hematology and Oncology, Emanate Health, West Covina, USA; 5 Family Medicine, Loma Linda University, Loma Linda, USA

**Keywords:** invasive aspergillus, immunocompetent patients, steroid use, covid-19, cns aspergilloma

## Abstract

*Aspergillus* is a ubiquitous saprophyte found in air, soil, and organic matter. Humans inhale the spore form of the fungus, but manifestations of the disease are typically predominantly seen in immunocompromised patients. Invasive central nervous system (CNS) aspergillosis is even more uncommon, and epidemiological data is sparse, particularly in immunocompetent patients.

We report the case of a 67-year-old previously immunocompetent female with no known comorbidities who was treated with corticosteroids for COVID-19 one month prior to admission for altered mental status (AMS). Subsequent imaging and biopsy demonstrated invasive CNS *Aspergillus niger*. Though a rare cause of AMS in immunocompetent patients, this report draws attention to the detrimental immunosuppressive effects of corticosteroid therapy in COVID-19.

## Introduction

*Aspergillus*, a fungus commonly found in the environment, is present in air, soil, and organic materials [1]. Among *Aspergillus* species, *Aspergillus fumigatus* accounts for roughly 60% of infections, with *Aspergillus flavus*, *Aspergillus niger*, and *Aspergillus terreus* following in frequency [1]. Diagnosis of *Aspergillus* is typically via growth on routine media. While humans routinely inhale its spores, the spectrum of disease can vary, including allergic symptoms, chronic pulmonary disease, and invasive disease [2]. Invasive *Aspergillus* infection typically manifests in individuals with compromised immune systems and has a high morbidity and mortality rate [2]. Invasive central nervous system (CNS) aspergillosis is particularly rare, and data on its epidemiology, especially in immunocompetent individuals, is limited. *Aspergillus* is treated with antifungals, and in some invasive cases, surgical intervention is necessary [3].

This case presents a 67-year-old previously immunocompetent woman with no underlying health conditions who received corticosteroid treatment for COVID-19 prior to experiencing altered mental status (AMS) and subsequent hospital admission. Imaging and biopsy revealed an invasive CNS infection caused by *Aspergillus niger*.

While AMS is an uncommon presentation of *Aspergillus* infection in immunocompetent individuals, this report underscores the concerning immunosuppressive effects of corticosteroid therapy, especially in the context of COVID-19 management.

## Case presentation

A 67-year-old female with a history of COVID-19 and no other known past medical or surgical history presented with AMS and an inability to open her eyes. Approximately one month prior to presentation, she was treated with dexamethasone (6 mg) intravenously for 10 days for COVID-19 pneumonia. On physical examination, the patient appeared somnolent, was nonverbal, and there were no identifiable rashes on her body. Upon further detailed neurologic examination, the patient demonstrated spontaneous movement of all four extremities, but she was unable to follow any commands. The patient was only responsive to painful stimuli, and her eyes and jaw were tightly shut.

**Figure 1:**
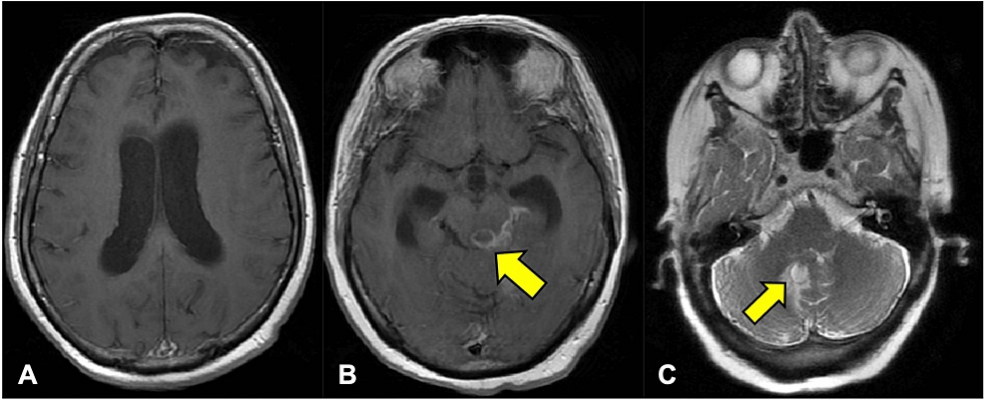
An MRI brain with contrast at the time of presentation demonstrates moderate to severe obstructive hydrocephalus (A) and a small rim-enhancing lesion in the left dorsal midbrain (yellow arrow in B). A subjacent signal abnormality, indicative of perilesional hemorrhage on additional sequences, is partially visualized. MRI brain with contrast five months post-diagnosis (C) shows a cystic focus within the right medial cerebellum, right posterior to the fourth ventricle (yellow arrow) MRI: magnetic resonance imaging

The initial workup included evaluation for infectious causes with a complete blood count, which revealed no abnormalities such as cytopenias. Additionally, a chest X-ray exhibited no evidence of post-COVID-19 lung changes, and urinalysis was within normal limits. Further workup for neurological causes of the AMS with magnetic resonance imaging (MRI) of the head showed moderate to severe hydrocephalus (Figure 1a) and a small rim-enhancing lesion within the left dorsal midbrain (Figure 1b). Further characterization of the rim-enhancing lesion was undergone via transnasal biopsy; histopathology revealed septate hyphae consistent with *Aspergillus niger*. Treatment with amphotericin B and isavuconazole was immediately started and later tailored to voriconazole. Despite aggressive antifungal therapy, the patient’s condition did not improve, prompting an endoscopic third ventriculostomy and a midline occipital craniotomy.

After continuation with voriconazole, a repeat MRI five months after the initial presentation showed stable moderate hydrocephalus, enhancement within the fourth ventricle, and a cystic focus within the right medial cerebellum (Figure 1c). Repeat hospitalizations were complicated by healthcare-associated pneumonia and urinary tract infections. Her neurologic status gradually worsened despite treatment with medications and therapy, leaving her bedridden, quadriplegic, and experiencing worsening lethargy and dysphagia.

## Discussion

*Aspergillus* is a ubiquitous saprophyte found in air, soil, and organic matter; humans inhale the spore form of the fungus, though the disease is typically seen only in immunocompromised patients. The prevalence of *Aspergillus* species has changed over time. Though *Aspergillus fumigatus* accounted for 90% of infections approximately ten years ago, it is now estimated to cause 60% of *Aspergillus* infections, followed in frequency by *Aspergillus flavus*, *Aspergillus niger*, and *Aspergillus terreus* [1].

Invasive aspergillosis is the most severe form of aspergillosis and occurs when the infection spreads from the lungs to other organs. Invasive aspergillosis is an uncommon infection that is most often seen in immunocompromised patients, such as those with AIDS, transplantation (including graft-versus-host disease in the setting of bone marrow transplantation), neutropenia, and prolonged corticosteroid use [4-7]. Invasive pulmonary aspergillosis is the presumed precipitating source for CNS infection, with hematogenous spread representing the dominant etiology, though direct or contiguous spread has also been described [8-12]. CNS *Aspergillus* is the second most common site of invasive disease after pulmonary infection, representing approximately 10-20% of invasive cases [3,13].

The extent of CNS invasion, location of intracranial spread, and clinical manifestations of *Aspergillus* differ through described cases in the literature, but focal neurological signs, fever, headache, vomiting, papilledema, seizures, and changes in mental status are reported symptoms at presentation [3], raising concern for meningitis, meningoencephalitis, or increased intracranial pressure [11].

Though precise incidence is difficult to assess, case series estimate incidence to be approximately one to two cases per 100,000 population [14]. CNS-invasive disease is associated with the highest mortality rate of all invasive aspergillosis syndromes, with up to 90% in most series [13]. The overall prognosis of intracranial *Aspergillus* is poor in both immunocompromised and immunocompetent patients [9].

Imaging in CNS aspergillosis can demonstrate sinonasal disease with intracranial extension (rhinocerebral disease), multiple or solitary mass-like parenchymal lesions, and/or meningeal involvement, with possible granuloma formation. Imaging appearances vary according to the site of involvement, but isolated mass-like parenchymal lesions are typically hypo- to iso-intense signals on T1-weighted and T2-weighted imaging, with heterogeneous peripheral enhancement on post-gadolinium imaging [1,9]. *Aspergillus* abscesses may also demonstrate peripheral low-signal intensity on T2-weighted images due to perilesional hemorrhage. Regions of restricted diffusion and decreased perfusion on perfusion-weighted imaging are also classic findings of fungal infection [15]. MRI findings in immunocompetent patients with CNS aspergillosis can include a thick, irregular wall of the mass, representing encapsulation of the infection by a competent immune system. Perilesional hemorrhage in the capsular wall in immunocompetent hosts is typically multifocal, rather than the more continuous T2 hypointense rim seen in immunocompromised patients [16].

Treatment for CNS infection depends on clinical presentation, medications that contraindicate treatment with amphotericin B and triazoles, and tolerance of these medications. Common treatment plans include a combination of surgical intervention and antifungal medication [3,17]. The most common surgical approach consists of a minimally invasive aspiration by the stereotaxic method or neuronavigation [3]. Though cases in the literature report successful treatment of intracranial *Aspergillus* utilizing voriconazole and a corticosteroid [17], outcomes in such patients remain guarded. A case study involving 81 patients who received a voriconazole treatment showcased that 31% of patients were alive and well following their diagnosis of intracranial *Aspergillus* one year later [8,18]. Though this 69% mortality rate remains exceedingly high, it has significantly decreased compared to the 85-100% reported generally for CNS *Aspergillus* [2]. Experimental treatment plans are currently being investigated and published in new and emerging case reports.

The implications of corticosteroid therapy in this patient’s case cannot be overstated. Corticosteroids started for COVID-19 pneumonia likely caused a transient immunocompromised state. Though corticosteroids have been shown to improve outcomes for COVID-19 patients with severe disease [19], the deleterious effects of such therapies must be balanced against the benefits for patients. It is important to highlight the guidelines surrounding low-dose and short-term application of corticosteroid therapy as reported by the RECOVERY trial [20]. Furthermore, consideration of transient immunocompromised states is critical in the current COVID-19 landscape. Further analysis of the unforeseen deleterious effects of corticosteroids is needed. In the meantime, including diagnoses that are typically only seen in immunocompromised patients on differential diagnoses in the era of COVID-19 allows clinicians to more quickly diagnose life-threatening conditions.

## Conclusions

Corticosteroid treatment for COVID-19 pneumonia likely predisposed our patient to invasive CNS *Aspergillus niger *in this case. When combined with hospitalization and microvascular changes due to COVID-19, a previously healthy woman was predisposed to a life-threatening disease with poor outcomes from the immunosuppressive effects of dexamethasone. The deleterious effects of corticosteroids are not typically discussed but should be considered in the era of COVID-19. Simultaneously, further research into the effects of corticosteroids on COVID-19 patients is needed to prevent late-stage diagnoses of life-threatening conditions.
